# Willingness to pay to avoid metastatic breast cancer treatment side effects: results from a conjoint analysis

**DOI:** 10.1186/2193-1801-3-350

**Published:** 2014-07-10

**Authors:** Deepa Lalla, Rashad Carlton, Eduardo Santos, Thomas Bramley, Anna D’Souza

**Affiliations:** Palo Alto Outcomes Research, Palo Alto, CA USA; Xcenda, Palm Harbor, FL USA; Genentech, South San Francisco, CA USA

**Keywords:** Willingness to pay, Conjoint analysis, Metastatic breast cancer

## Abstract

**Purpose:**

Metastatic breast cancer (MBC) patients are treated with a variety of regimens with differing side effects that can reduce the patients’ quality of life. This study assessed the willingness to pay (WTP) to avoid side effects related to MBC treatment using conjoint analysis.

**Methods:**

An online, self-administered conjoint analysis survey of US adult female MBC patients was conducted to elicit preferences for MBC treatment side effects. Attributes included in the analysis were hair loss, diarrhea, fatigue, nausea, tingling in hands and feet, pain, risk of infection, and out-of-pocket costs. Fifteen choice-based conjoint questions were presented where patients selected the most preferred therapy. A partial profile design was used to allow for each treatment description to be made with 3 instead of all 8 attributes. The attribute choices for each question included 2 side effects and a yearly out-of-pocket price.

**Results:**

There were 298 respondents. MBC patients were willing to pay (US$) $3,894 to avoid severe diarrhea, $3,479 to avoid being hospitalized due to infection, $3,211 to avoid severe nausea, $2,764 to avoid severe tingling in hands and feet, $2,652 to avoid severe fatigue, $1,853 to avoid obvious hair loss, and $1,458 to avoid severe pain. The most important attributes when selecting a therapy for MBC in terms of average utility were risk of infection, diarrhea, and nausea.

**Conclusions:**

MBC patients were willing to pay significant amounts to avoid side effects associated with MBC treatment, with patients willing to pay the most to avoid diarrhea, risk of infection, and nausea.

## Introduction

Breast cancer is the most frequently diagnosed cancer in women after skin cancer. It is estimated that 226,870 women will be diagnosed with and 39,150 women will die of breast cancer in 2012 (Howlader et al. 2012). The overall 5-year relative survival for breast cancer from 2002 to 2008 was 89.0%. The majority of women with breast cancer will present with the disease localized to the primary site (60%) or with spread to regional lymph nodes (33%), where the 5-year relative survival is over 80% (Howlader et al. 2012). Approximately 5% of women with breast cancer present with more severe metastatic disease at diagnosis (Howlader et al. 2012). The 5-year survival rate for women with metastatic breast cancer (MBC) at diagnosis is only 23.8% (Howlader et al. 2012). Newer chemotherapy agents and hormone agents used for the treatment of MBC have led to significant improvements in patients’ survival (Pal et al. 2012; Andre et al. 2004; Chia et al. 2007). As survival improves, patients are being exposed to therapy for longer periods of time, and quality of life optimization is a goal of treatment. Agents used in the treatment of MBC may have associated side effects that can affect and reduce quality of life for patients with MBC (Romond et al. 1995; Piccart-Gebhart et al. 1995; Shapiro and Recht 2001; Tannock et al. 1998; Osaba et al. 2003). These associated side effects, which vary by agent, can be an important consideration when evaluating the best regimen for a patient.

Lindley et al. showed that women who experience severe disruptions in quality of life are less willing to receive additional treatment for an extension of life by 6 months compared with women who experienced no or little disruption in normal life (Lindley et al. 1998). Assessing the perceived value of a lower risk of toxicity provides additional information on how patients view the tolerability of chemotherapy agents, potentially assisting in the selection of preferred agents. The objective of this analysis was to assess the importance of MBC treatment side effects and to assess the willingness to pay (WTP) to avoid these side effects.

## Methods

A survey was developed using conjoint analysis to elicit preferences or utilities for treatments for MBC based on the side-effect profile of the treatment. Conjoint analysis involves comparing hypothetical scenarios by ranking, rating, or choosing a particular scenario (Phillips et al. 2002). Conjoint analysis elicits preferences by asking respondents to evaluate alternatives consisting of different combinations of attributes (Phillips et al. 2002). Respondent choices indicate the relative importance of the product attributes and provide data for estimating utilities. The conjoint analysis technique is based on economic theory and the assumption that individuals maximize a preference (or utility) function (Phillips et al. 2002). Conjoint analysis can also be used to estimate how individuals trade between attributes; for example, the rate at which they are willing to give up one unit of an attribute for an increase in another attribute (Ryan 1999). This is known as the marginal rate of substitution (MRS). When applied to the current study, treatments for MBC were defined in terms of their side-effect profile, with the side effects constituting the attributes of the treatment. The conjoint analysis technique was then used to obtain utilities for each of the treatment-related side effects.

The conjoint analysis technique consists of 5 steps (Ryan and Farrar 2000). First, the attributes are defined. Seven side effects of MBC treatments were chosen as attributes for the survey. The side effect attributes were hair loss, fatigue, nausea, pain, diarrhea, risk of infection, and tingling in hands and feet. Additionally, cost was included as an attribute to allow for calculation of the WTP to avoid side effects. Second, the levels for each of the attributes are set. Levels for the attributes were based on severity of mild/low, moderate/medium, or severe/high (Table 1). Attributes and levels were selected based on the literature and in collaboration with clinicians based on the side effects and severity levels commonly seen in clinical practice when treating MBC patients. Attributes were described in non-medical, lay terminology so that they could be easily understood by patients (eg, “hair loss” instead of “alopecia”).

**Table 1:** **Attributes and levels of side effects**

Attribute	Level of severity		
	Mild/Low	Moderate/Medium	Severe/High
Hair loss	None/not noticeable	--	Obvious
Fatigue	None/full activity	I often need a nap to reset myself	Major impact on my activity level
Nausea	None/easy to ignore	Manageable with medication/still eating	Can’t eat
Pain	None/easy to ignore	Manageable with OTC medication (eg, Advil, Tylenol)	Need prescription-strength medication
Diarrhea	2 stools or less/day	3+ loose stools per day	Unable to leave the house due to frequency and urgency of diarrhea
Risk of infection	None/modest	--	Hospitalized due to infection
Tingling in hands and feet	None/easy to ignore	Bothersome but manageable	Interferes with activities of daily living (eg, getting dressed)
Out-of-pocket cost	$500	$1,000	$3,000

Key: OTC – over-the-counter.

Third, scenarios are created. The 8 attributes in the analysis, each with 2 or 3 levels, give rise to 2,916 possible combinations (3^6^ × 2^2^ = 2,916). It is implausible to assess the utility for each respondent on all attributes and levels with such a large number of possible combinations. An orthogonal main effects design was therefore used to reduce the number of possible combinations to a manageable level while still being able to infer utilities for all possible scenarios (Ryan 1999). The orthogonal design resulted in the creation of 15 survey versions for each respondent. Previous research suggests that individuals can manage between 9 and 16 pairwise comparisons before they become tired or bored (Pearmain et al. 1991). Additionally, a partial profile design was chosen over a full-profile design that allowed for treatment descriptions to be made with 3 instead of all 8 attributes (Ryan and Farrar 2000). Each treatment choice was described in terms of 2 side effects (randomly chosen) and an out-of-pocket cost level.

Fourth, preferences are established (Ryan and Farrar 2000). In this analysis, we employed the discrete-choice method where respondents were presented with 3 treatment choices for each conjoint question and were asked to choose their preferred treatment. The discrete choice approach was preferred as it mimics the manner in which decisions are made in real life and is based on random utility theory (Ratcliffe 2000). The final step in the conjoint analysis process is data analysis (Ryan and Farrar 2000). The random effects multinomial logit model in Sawtooth software was used to analyze the data. Effects coding was used to scale the sum to zero within each attribute for determining conjoint utilities/value to the patient (Orme 2010). The relative importance of the side effects was obtained by averaging the absolute values of the attribute level coefficients across levels of attributes, including the baseline coefficients. WTP was estimated as the amount respondents were willing to pay to avoid each side effect level and have a baseline level of no or minimal side effect. This was calculated by dividing the coefficient differences between the baseline level of no or minimal side effect and the respective side-effect level by the coefficient for out-of-pocket cost (Orme 2010).

The survey was fielded as an online, self-administered survey to MBC patients. Patients were identified from a US consumer panel of breast cancer patients and were sent an email link to complete the survey. Respondents were compensated (gift certificate, <$25) for their time for completing the survey. Survey respondents completed 15 conjoint analysis questions in addition to background demographic questions and questions on their history of side effects while on MBC treatments.

## Results

There were a total of 298 respondents. The majority of respondents were white (84%), married (57%), over 40 years old (86%), and had private insurance (57%). Respondents were evenly distributed across the country and mostly resided in suburban areas (53%). Most respondents had a degree (associates or higher) and a household income above $50,000 (53%) (Table 2).

**Table 2:** **Description of study sample**

Characteristic	Percent of respondents (n = 298)
*Insurance Status*	
Private insurance	57.1%
Medicare	13.8%
Medicare w/supplemental	14.8%
Medicaid	7.4%
Veterans Affairs	1.3%
Other	5.7%
*Marital Status*	
Single	18.5%
Married	57.5%
Divorced	18.8%
Widowed	5.5%
*Race*	
White	83.9%
Black	9.1%
Asian	1.3%
Hispanic	4.4%
Other	1.3%
*Region*	
Northeast	28.9%
Midwest	25.5%
South	23.5%
West	22.1%
*Area of Residence*	
Rural	20.5%
Suburban	53.0%
Mid-sized urban	10.1%
Urban	16.4%
*Education Level*	
Some high school	3.0%
High school	21.8%
Trade school	12.1%
Associate degree	19.5%
Bachelor degree	27.5%
Graduate degree	16.1%
*Age*	
30 years or younger	3.0%
31–40 years	21.8%
41–50 years	12.1%
51–60 years	19.5%
61–70 years	27.5%
71+ years	16.1%
*Household Income*	
Under $10,000	7.1%
$10,000–$25,000	13.4%
$26,000–$50,000	26.5%
$51,000–$75,000	23.5%
$76,000–$100,000	14.4%
Over $100,000	15.1%

### Metastatic breast cancer experience

**Figure 1:**
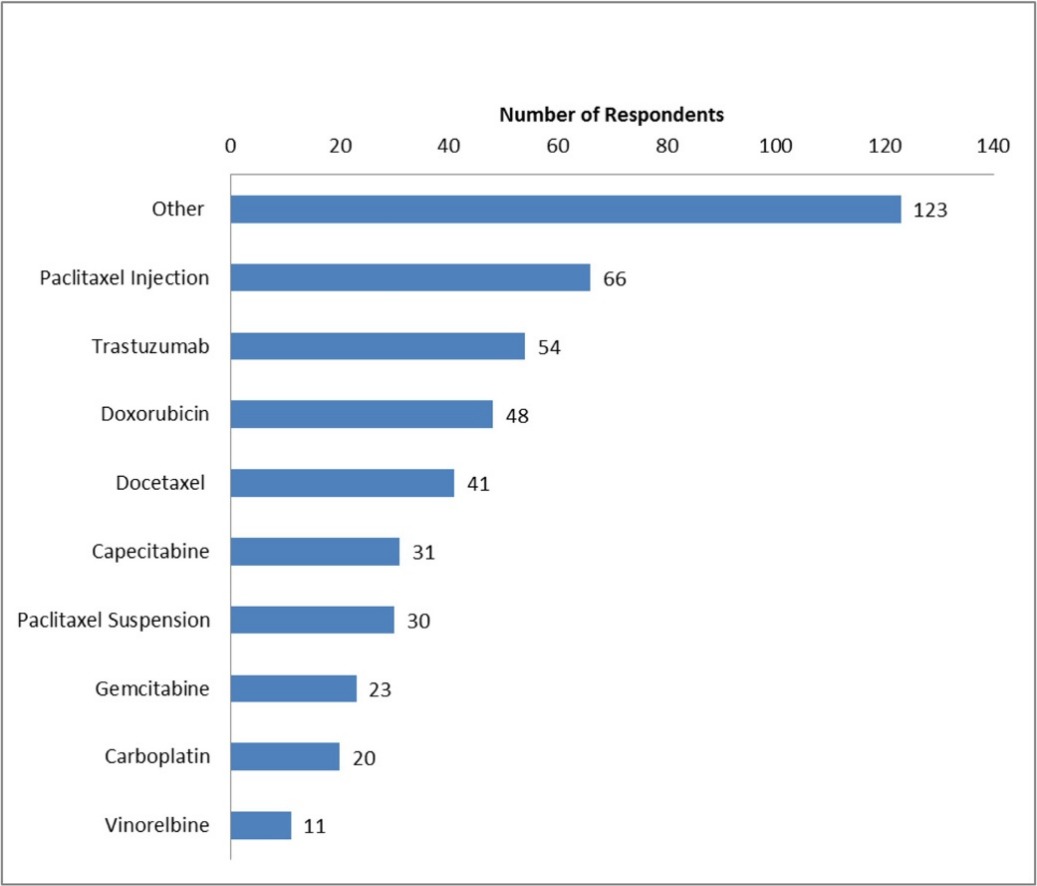
**Current or most recent treatment regimen.** Other chemotherapy treatments reported were letrozole, zoledronate, ixabepilone, cyclophosphamide, fulvestrant, anastrozole, tamoxifen, exemestane, denosumab, mitomycin, eribulin, leuprolide, pamidronate, lapatinib, raloxifene, ibandronate.

**Figure 2:**
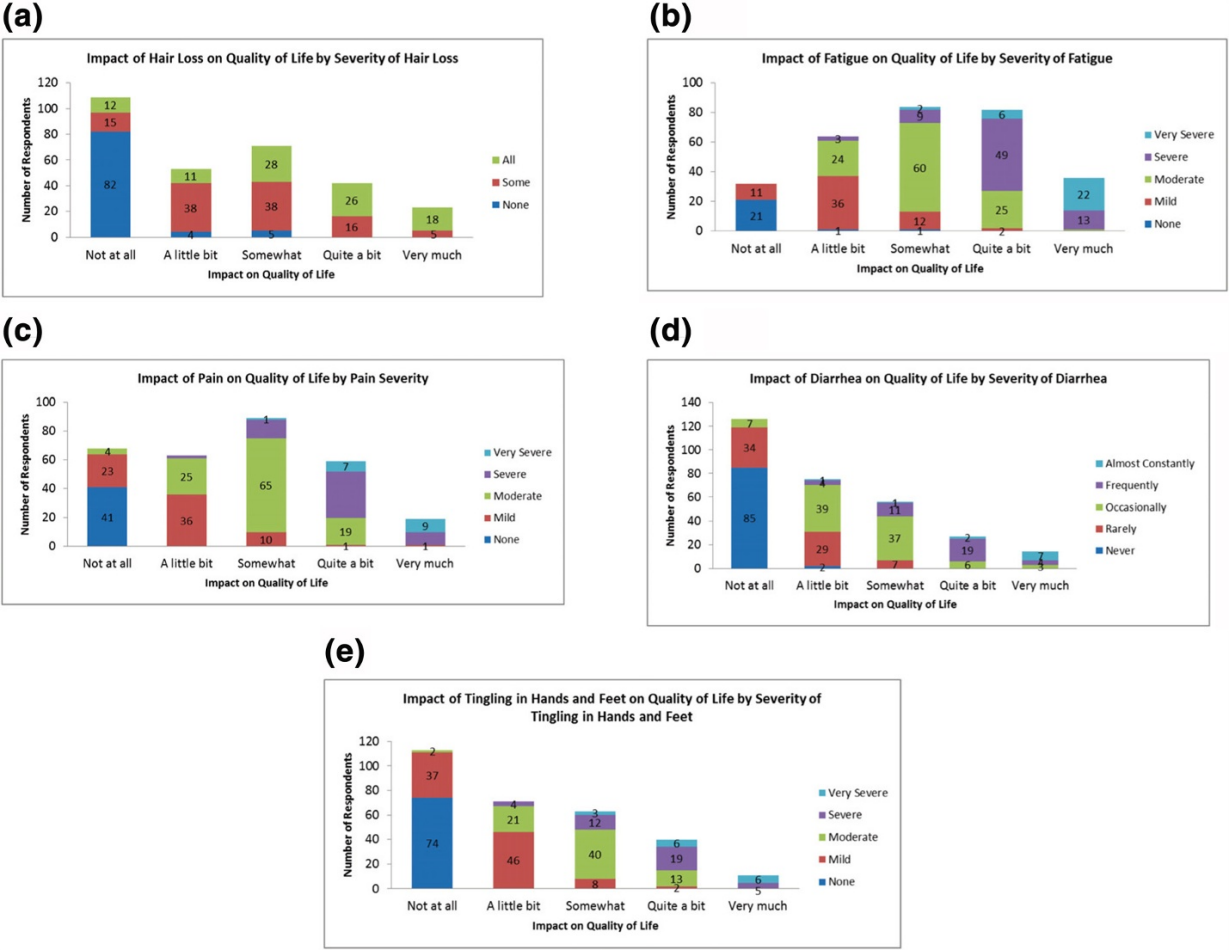
**Impact of side effects on quality of life by Severity of Side Effects. (a)** Hair loss **(b)** Fatigue **(c)** Pain **(d)** Diarrhea **(e)** Tingling in hands and feet.

Approximately 71% of patients were receiving treatment for MBC at the time of the survey. The most common chemotherapies mentioned as part of their current regimen included paclitaxel, trastuzumab, doxorubicin, anastrozole, and cyclophosphamide (Figure 1). The average out-of-pocket cost reported by the respondents for their last regimen was $832. Most respondents (i) experienced moderate fatigue; (ii) lost none or only some of their hair, and (iii) experienced mild to moderate nausea, pain, diarrhea, and tingling of hands and feet. The impact of side effects on quality of life was closely related to severity of adverse events. As respondents experienced more severe side effects, they reported a more negative quality of life (Figure 2).

### Willingness to pay

**Figure 3:**
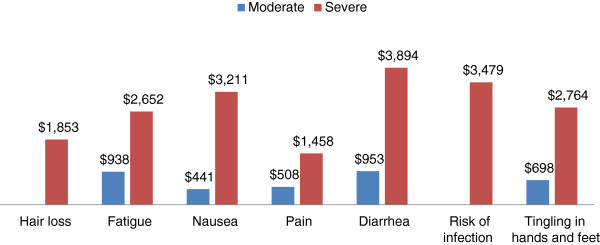
**Average yearly willingness to pay for a reduction in treatment-related side effects by Severity of Side Effects.**

Conjoint analysis was used to determine the maximum yearly total out-of-pocket amount that respondents would be willing to pay for an entire course of therapy that would provide equal effectiveness and a reduction in treatment-related side effects. WTP serves as a proxy of patient utility by measuring patients’ desire to avoid side effects that can negatively affect the treatment’s value to the patient. The side effects that patients were willing to pay the most to avoid were diarrhea so severe they could not leave the house ($3,894), being hospitalized due to an infection ($3,479), nausea so severe that they could not eat ($3,211), and tingling in the hands and feet that interferes with daily activities ($2,764) (Figure 3).

### Value to patient

The average value to patients (part-worth utility) for each attribute was calculated using effects coding. The relative importance of each attribute was determined based on the range of each attribute’s utility values. The attributes with the most utility to patients were risk of infection (1.0090), diarrhea (0.8809), and nausea (0.7709) (Table 3).

**Table 3:** **Average value to patient of a reduction in side effects**

Attribute	Levels	Value to patient	Average value to patient	Importance
Hair loss	None/not noticeable	0.5374	0.5374	6
	Obvious	-0.5374		
Fatigue	None/full activity	0.6941	0.5628	5
	I often need a nap to reset myself	0.1501		
	Major impact on my activity level	-0.8841		
Nausea	None/easy to ignore	0.7060	0.7709	3
	Manageable with OTC medication (eg, Advil, Tylenol)	0.0854		
	Need prescription-strength medication	-0.4654		
Diarrhea	2 stools or less per day	0.9371	0.8809	2
	3+ loose stools per day	0.3842		
	Unable to leave the house due to frequency and urgency of diarrhea	-1.3213		
Risk of infection	None/modest	1.090	1.090	1
	Hospitalized due to infection	-1.090		
Tingling in hands and feet	None/easy to ignore	0.6693	0.6624	4
	Bothersome, but manageable	0.2643		
	Interferes with daily activities (eg, getting dressed)	-0.9336		

The average value to patients is the part-worth utility calculated using effects coding. The relative importance of each attribute was determined based on the range of each attribute’s utility values.

## Discussion

The results of the analysis indicate that patients were willing to pay over $3,000 a year to avoid severe diarrhea, being hospitalized due to infection, and severe nausea. The most important attribute based on utility to patients in treatment decisions was the risk of infection/febrile neutropenia. In previous conjoint analysis research in patients with breast cancer, neutropenia with hospitalization, nausea, and fatigue were found to have the most impact on patients’ preferences for chemotherapy (Beusterien et al. 2012). The results of this analysis are consistent with previous patient preference research using conjoint analysis. Previous research on the health utilities of MBC side effects has shown that febrile neutropenia/risk of infection has a major impact on quality of life (utility -0.150) (Lloyd et al. 2006). The utility for febrile neutropenia was higher than the utility for other side effects such as hand-foot syndrome (-0.116), fatigue (-0.115), hair loss (-0.114), and diarrhea/vomiting (-0.103) (Lloyd et al. 2006). The utilities of attributes from this analysis are consistent with previous research on the utilities of MBC, with febrile neutropenia having the largest impact on quality of life (Lloyd et al. 2006). In our analysis, diarrhea had higher utility to patients than in previous research. This may be driven by the conjoint methodology and the distribution of levels of diarrhea, with the most severe level of diarrhea leaving the patient in a state where they are unable to leave the house due to the frequency and urgency of diarrhea.

The results elicited in this analysis using conjoint analysis are different from those seen when patients are asked directly which side effect they would most like to avoid. In a previous survey of 202 MBC patients, the side effects patients responded that they were most likely to pay extra to avoid were hair loss (28.2%), pain (16.8%), and nausea (14.9%) (Lalla et al. 2011). In the previous survey, patients assessed the importance of each side effect individually without reference to price or the severity of the side effect and without any tradeoffs between attributes. When presented with conjoint questions looking at different scenarios and faced with tradeoffs between attributes, hair loss and pain went from 2 of the most important attributes to the least 2 important attributes in a conjoint analysis.

When presented with an open-ended question to assess how much they would be willing to pay for a 25%, 50%, or 100% reduction in MBC treatment side effects, respondents were willing to pay $1,886 for a 25% reduction in MBC treatment side effects, $3,837 for a 50% reduction in MBC treatment side effects, and $7,794 for a 100% reduction in MBC treatment side effects (Lalla et al. 2011). In the previous survey, patients were willing to pay more to avoid treatment-related side effects when presented with an open-ended response. The difference in the WTP estimates between this study and Lalla et al. (2011) is likely due to the discrete choice framework approach used in the current study, where respondents are effectively locked into the choice between the scenarios presented (Ratcliffe 2000). For example, in our survey, the lowest out-of-pocket cost was $500. The respondent may only be willing to pay $300 for the scenarios under consideration. The respondent is unable to give his/her true preference as their WTP amount is not a given option. Similarly, the highest out-of-pocket cost in our survey was $3,000. There may be some patients who are willing to pay more than $3,000 for one of the scenarios, and thus they are unable to give their true willingness to pay value.

### Limitations

This analysis has a number of limitations. First, these results do not specifically correlate WTP with insurance status or other determinants of out-of-pocket costs. Patients were asked to choose scenarios based on the yearly out-of-pocket costs; however, patient factors such as insurance status, socioeconomic background, income level, and comorbid diseases may affect the willingness and ability to pay for MBC treatments. Finally, results from this survey may not be generalizable to the entire MBC patient population. This survey population represents a small subset of the MBC patient population who were healthy enough and willing to complete a 30-minute online survey.

## Conclusions

Patients highly value reductions in side effects associated with MBC treatment and are willing to pay higher out-of-pocket costs to avoid the side effects that cause the largest reduction in quality of life. The side effects that had the most value or utility to respondents were risk of infection, diarrhea, and nausea. Although risk of infection was considered by respondents to be the most important side effect, as measured by the average utilities, survey respondents had the highest WTP for avoidance of severe diarrhea. Understanding patients’ perspectives and preferences on which side effects they most wish to avoid can aid value-based decision making when selecting between MBC treatment options.

## References

[CR1] Andre F, Slimane K, Bachelot T, Dunant A, Namer M, Barrelier A, Kabbaj O, Spano JP, Marsiglia H, Rouzier R, Delaloge S, Spielmann M (2004). Breast cancer with synchronous metastases: trends in survival during a 14-year period. J Clin Oncol.

[CR2] Beusterien K, Grinspan J, Tencer T, Brufsky A, Visovsky C (2012). Patient preference for chemotherapies used in breast cancer. Int J Womens Health.

[CR3] Chia S, Speers C, D’yachkova Y, Kang A, Malfair-Taylor S, Barnett J, Coldman A, Gelmon K, O’Reilly S, Olivotto I (2007). The impact of new chemotherapeutic and hormone agents on survival in a population-based cohort of women with metastatic breast cancer. Cancer.

[CR4] Howlader N, Noone AM, Krapcho M, Neyman N, Aminou R, Altekruse SF, Kosary CL, Ruhl J, Tatalovich Z, Cho H, Mariotto A, Eisner MP, Lewis DR, Chen HS, Feuer EJ, Cronin KA (2012). SEER Cancer Statistics Review, 1975–2009 (Vintage 2009 Populations).

[CR5] Lalla D, McLaughlin T, Brammer M, Bramley T, Bare A, Carlton R (2011). Presented at the ISPOR 14th Annual European Congress, November 5–8, 2011; Madrid, Spain. Willingness to pay for a reduction in the risk of treatment-related side effects in patients with metastatic breast cancer [poster].

[CR6] Lindley C, Vasa S, Sawyer WT, Winer EP (1998). Quality of life and preferences for treatment following systemic adjuvant therapy for early-stage breast cancer. J Clin Oncol.

[CR7] Lloyd A, Nafees B, Narewska J, DeWilde S, Watkins J (2006). Health state utilities for metastatic breast cancer. Br J Canc.

[CR8] Orme B (2010). Getting started with conjoint analysis: strategies for product design and pricing research.

[CR9] Osaba D, Slamon D, Burchmore M, Murphy M (2003). Effects on quality if life of combined trastuzumab and chemotherapy in women with metastatic breast cancer. J Clin Oncol.

[CR10] Pal S, Dehaven M, Nelson R, Onami S, Hsu J, Waliany S, Kruper L, Mortimer J (2012). Impact of modern chemotherapy on the survival of women presenting with de novo metastatic breast cancer. BMC Cancer.

[CR11] Pearmain D, Swanson J, Kroes E, Bradley M (1991). Stated preference techniques: a guide to practice.

[CR12] Phillips K, Johnson R, Maddala T (2002). Measuring what people value: a comparison of attitude and preference surveys. Health Serv Res.

[CR13] Piccart-Gebhart MJ, Procter M, Leyland-Jones B, Goldhirsch A, Untch M, Smith I, Gianni L, Baslega J, Bell R, Jackish C, Cameron D, Dowsett M, Barrios C, Steger G, Huang C, Andersson M, Inbar M, Lichinster M, Nitz U, Iwata H, Thomassen C, Lohrisch C, Suter T, Ruschoff J, Suto T, Greatorex V, Ward C, Straehle C, McFadden E, Dolci S (1995). Trastuzumab after adjuvant chemotherapy in HER2-positive breast cancer. N Engl J Med.

[CR14] Ratcliffe J (2000). The use of conjoint analysis to elicit willingness-to-pay values. Int J Technol Assess Health Care.

[CR15] Romond EH, Perez EA, Bryant J, Suman V, Geyer C, Davidson N, Tan-Chiu E, Martino S, Paik S, Kaufman P, Swain S, Pisansky T, Fehrenbacher L, Kutteh L, Vogel V, Visscher D, Yothers G, Jenkins R, Brown A, Dakhil S, Mamounas E, Lingle W, Klein P, Ingle J, Wolmark N (1995). Trastuzumab plus adjuvant chemotherapy for operable HER2-positive breast cancer. N Engl J Med.

[CR16] Ryan M (1999). Using conjoint analysis to take account of patient preferences and go beyond health outcomes: an application to in vitro fertilization. Soc Sci Med.

[CR17] Ryan M, Farrar S (2000). Using conjoint analysis to elicit preferences for health care. BMJ.

[CR18] Shapiro CL, Recht A (2001). Side effects of adjuvant treatment of breast cancer. N Engl J Med.

[CR19] Tannock IF, Boyd NF, DeBoer G, Erlichman C, Fine S, Larocque G, Mayers C, Perrault D, Sutherland H (1998). A randomized trial of two doses of cyclophosphamide, methotrexate, and fluorouracil chemotherapy in patients with metastatic breast cancer. J Clin Oncol.

